# Regulation of *AtKUP2* Expression by bHLH and WRKY Transcription Factors Helps to Confer Increased Salt Tolerance to *Arabidopsis thaliana* Plants

**DOI:** 10.3389/fpls.2020.01311

**Published:** 2020-08-25

**Authors:** Sivamathini Rajappa, Pannaga Krishnamurthy, Prakash P. Kumar

**Affiliations:** ^1^Department of Biological Sciences, National University of Singapore, Singapore, Singapore; ^2^NUS Environmental Research Institute (NERI), National University of Singapore, Singapore, Singapore

**Keywords:** salt tolerance, mangrove, KUP2, bHLH, WRKY, ChIP

## Abstract

Potassium transporters play an essential role in maintaining cellular ion homeostasis, turgor pressure, and pH, which are critical for adaptation under salt stress. We identified a salt responsive *Avicennia officinalis* KUP/HAK/KT transporter family gene, *AoKUP2*, which has high sequence similarity to its *Arabidopsis* ortholog *AtKUP2*. These genes were functionally characterized in mutant yeast cells and *Arabidopsis* plants. Both *AoKUP2* and *AtKUP2* were induced by salt stress, and *AtKUP2* was primarily induced in roots. Subcellular localization revealed that AoKUP2 and AtKUP2 are localized to the plasma membrane and mitochondria. Expression of *AtKUP2* and *AoKUP2* in *Saccharomyces cerevisiae* mutant strain (BY4741 *trk1*Δ*::loxP trk2*Δ*::loxP*) helped to rescue the growth defect of the mutant under different NaCl and K^+^ concentrations. Furthermore, constitutive expression of *AoKUP2* and *AtKUP2* conferred enhanced salt tolerance in *Arabidopsis* indicated by higher germination rate, better survival, and increased root and shoot length compared to the untreated controls. Analysis of Na^+^ and K^+^ contents in the shoots and roots showed that ectopic expression lines accumulated less Na^+^ and more K^+^ than the WT. Two stress-responsive transcription factors, bHLH122 and WRKY33, were identified as direct regulators of *AtKUP2* expression. Our results suggest that AtKUP2 plays a key role in enhancing salt stress tolerance by maintaining cellular ion homeostasis.

## Introduction

Salinity is a major environmental threat for crop production because high concentration of salt in the soil severely affects plant performance by disturbing cellular metabolism. Such adverse effects of increased salinity occur mainly due to the osmotic stress and continuous accumulation of toxic ions within the plant cells. Additionally, this can lead to oxidative stress and nutritional imbalance (Munns and Tester, 2008). Ion transport is a critical step controlling ion homeostasis in plants. This includes the transport of ions across the root cell plasma membrane or through vacuolar membrane and also occurs *via* xylem and phloem in order to facilitate the accumulation and excretion of salt by specialized cells (Volkov and Beilby, 2017). The physiological and morphological disturbances under saline conditions are often caused by the accumulation of toxic ions such as Na^+^ and Cl^-^. This ion toxicity can be minimized by three possible ways, (i) compartmentalization of Na^+^ and Cl^-^ ions into the vacuole (ii) through exclusion of Na^+^ and Cl^-^ outside the cell and (iii) through limiting net Na^+^ and Cl*^−^* uptake (Munns and Tester, 2008; Roy et al., 2014).

The disproportionate accumulation of Na^+^ in the intracellular and extracellular compartments adversely affects the uptake and homeostasis of essential nutrients such as, K^+^ and Ca^2+^. The activation of Na^+^ and K^+^ transport systems helps in retaining the cellular ion homeostasis by maintaining low Na^+^ content in leaves and high K^+^/Na^+^ ratio in cytosol. The optimum maintenance of K^+^/Na^+^ ratio in cytosol can be achieved by either limiting the accumulation of Na^+^ in plant tissues or by preventing the loss of K^+^ across the plasma membrane (Almeida et al., 2017; Assaha et al., 2017). Thus far, most of the plant breeders and physiologists have focused mainly on the former mechanism. There is increasing evidence to show that the ability of a plant to maintain high K^+^/Na^+^ ratio in cytosol seems to be crucial for plant survival under salt stress. Rather than vacuolar content, the cytosolic K^+^ homeostasis is crucial for plant metabolic processes (Wu et al., 2018).

There are mainly two kinds of transport systems in plants, (a) through channels or (b) *via* transporters (Adams and Shin, 2014; Ruiz-Lau et al., 2016). Four multi-gene K^+^ transporter families are found in plants, (i) KUP/HAK/KT, (ii) HKT/Trk, (iii) CHX, and (iv) KEA transporters (Véry and Sentenac, 2003; Sharma et al., 2013; Yang et al., 2014; Gupta et al., 2018). The large family of high affinity potassium transporters (HAK/KUP/KT) in plants mainly contribute to root K^+^ acquisition under a wide range of external K^+^ levels. They also mediate K^+^ movement within the plant and K^+^ efflux into the environment, thereby maintaining ion homeostasis (Liang et al., 2020). Among these transporters, HAKs have been extensively studied. In rice, induction of *OsHAK1* expression was observed upon NaCl treatment or by K^+^ deficiency (Chen et al., 2015). Constitutive expression of *OsHAK5* in BY2 tobacco cells improved the K^+^ accumulation but not the Na^+^ accumulation under salt treatment and conferred increased salinity tolerance (Horie et al., 2011). Most of the AtKT/KUPs are known to be localized to the root hairs and root tip (Ahn et al., 2004). The physiological functions in relation to K^+^ transportation, acquisition, and improving salinity tolerance of other distinct members of plant KUP/KT/HAK transporter family have not been well studied. Maintenance of K^+^ uptake under high external Na^+^ is crucial for K^+^/Na^+^ homeostasis leading to salt tolerance (Munns and Tester, 2008). On the other hand, increased Na^+^ level suppresses the expression of several HAK/KUP/KT K^+^ transporters at low K^+^ conditions (Nieves-Cordones et al., 2010). Therefore, in order to devise strategies for crop improvement, it is important to identify and characterize KUP transporters that can function under elevated NaCl conditions.

The KUP/HAK/KT transporter family was first identified in bacteria through mutagenesis of K^+^-dependent *Escherichia coli* strains harboring the *kdpABC5* mutation (Epstein and Kim, 1971). The *KUP* family has 13 genes in *Arabidopsis* and 27 genes in rice. Their ubiquitous presence in plants suggests that they play a significant role in acquisition of nutrients and survival in potassium-poor environments. In *Arabidopsis*, it was shown that AtKUPs (KUP1, 2, 4, 5-7, 10, and 11) complement a mutant *E. coli* strain that lacks K^+^ uptake gene, which proves that these KUP proteins play an important function in transport of K^+^ (Fu and Luan, 1998; Kim et al., 1998; Ahn et al., 2004; Gierth and Mäser, 2007; Horie et al., 2011; Chen et al., 2015; Han et al., 2016). In *Arabidopsis*, the semi-dominant mutant of *AtKUP2* also named as *shy3-1*causes growth-related defects such as short hypocotyl, short flowering stem and small leaves (Elumalai et al., 2002). Since potassium is a main solute, defect in cellular homeostasis of K^+^ might affect the turgor pressure and perturb the expansion rate of cells. Increased expression of KUP members has shown contrasting results. While overexpression of *GhKT1* and *VvKUPs* (*VvKUP1* and *VvKUP2*) was shown to increase the turgor pressure to drive rapid elongation of cotton fibres and *Vitis vinifera*, respectively (Ruan et al., 2001; Davies et al., 2006), enhanced cell expansion was observed in the triple mutant *kup268* where the expression of *KUP2, KUP6* and *KUP8* are disrupted along with guard cell outward rectifying K^+^ channel gene, *GORK* (Osakabe et al., 2013). *KUP2, KUP6* and *KUP8* together are shown to have a role in lateral root (LR) formation by positively affecting the ABA and osmotic stress responses. Also, mutation of KUP leads to enhanced expression of auxin responsive *LBD* genes, which are involved in control of lateral root formation (Okushima et al., 2007; Lee et al., 2009). Altogether, *KUP2*, *KUP6*, and *KUP8* are involved in initiation of lateral root formation and in the development of antagonistic signal crosstalk between auxin and ABA. Similarly, AtKUP4 and AtKUP9 contribute to auxin homeostasis in Arabidopsis root. The disruption of AtKUP4/TRH1 (Tiny Root Hair 1) affects the root hair elongation due to auxin deficiency and AtKUP9 mediates K^+^ and auxin efflux to maintain meristem activity under low K^+^ stress (Rigas et al., 2001; Rigas et al., 2013; Daras et al., 2015; Zhang et al., 2020). This further highlights the functional importance of this gene family and the need to better understand them in plants.

Regulation of genes invariably involves specific transcription factors (TFs). Hence, in order to understand the molecular regulatory mechanisms behind the expression of *KUPs* and how they function in salinity stress tolerance, the associated TFs should be studied. Plants have developed complicated stress response strategies that include differential expression of genes encoding TFs. Various TFs such as bHLH, WRKY, MYC, NAC, MYB, and ERF/AP2 have been identified to be associated with salt tolerance mechanism (Okushima et al., 2007; Golldack et al., 2011; Hoang et al., 2017; Meraj et al., 2020). Nevertheless, information on how these TFs modulate their respective downstream target genes is quite limited in this area.

One of the approaches used to address salinity stress is through the investigation of salt responsive genes in halophytic species. Halophytes employ three mechanisms to confer salt tolerance; compartmentalization, reduction of the Na^+^ influx, and excretion of Na^+^ ions (Mishra and Tanna, 2017). In attempts to better understand the molecular mechanisms behind salinity tolerance, salt-responsive genes have been isolated from specific halophytes and expressed in glycophytes (Himabindu et al., 2016). *Avicennia officinalis* is a halophyte with unique characteristics such as efficient salt filtration at the roots and salt secreting glands on leaves (Krishnamurthy et al., 2017). However, not much work is done to understand the functioning of their ion transporters which might serve as potential candidates for generating salt tolerant crops.

In this report, the physiological function and expression patterns of *AoKUP2* from *A. officinalis* and its *Arabidopsis* ortholog, *AtKUP2* were characterized*. AoKUP2* and *AtKUP2* expression were studied in *Arabidopsis* under varying salt stress conditions. We show that expression of *AtKUP2* and *AoKUP2* in transgenic *Arabidopsis* and yeast improved growth, K^+^ uptake and salinity tolerance. Our findings show that *AtKUP2* expression is directly regulated by bHLH122 and WRKY33 while playing an essential function in mediating K^+^ transport and maintaining plant growth under salt stress.

## Materials and Methods

### Plant Materials and Growth Conditions (*Arabidopsis* and *A. officinalis*)

T-DNA insertional mutants, *atkup2* (SAIL_504_A07)*, atbhlh122* (SALK_002286), *atwrky33* (SALK_064436) and wild-type (WT) *Arabidopsis thaliana*, ecotype Columbia-0 were obtained from the Arabidopsis Biological Resource Center (ABRC) seed stock. Ectopic expression lines, *35S*::*AoKUP2* and *35S*::*AtKUP2* were generated by us for this study. *A. officinalis* L. (*A. officinalis*) propagules were collected and salt treated as described in (Krishnamurthy et al., 2014; Krishnamurthy et al., 2017).

### Cloning and Generation of Transgenic *Arabidopsis* Lines

Mutant line *atkup2* (SAIL_504_A07) with T-DNA insertion was obtained from the SALK collection (Alonso et al., 2003). Positions of T-DNA insertion sites are shown in **Supplementary Figure S1A**. Plants homozygous for the T-DNA insertion were selected by genotyping with primers designed using the T-DNA primer design tool (http://signal.salk.edu/tdnaprimers.2.html) (**Supplementary Figure S1B****)**. To check the suppression of *AtKUP2* in mutant, qRT-PCR was carried out. Seeds were collected from only those lines that showed more than 80% suppression of *AtKUP2* (**Supplementary Figure S1C****)**. For generation of ectopic expression lines in *Arabidopsis*, coding DNA sequences (CDS) of *AoKUP2* and *AtKUP2* were cloned into pGreen binary vector. The constructs *35S::AoKUP2, 35S::AtKUP2* and *pAtKUP2::GUS* were electroporated into *Agrobacterium tumefaciens* strain GV3101:pMP90 and introduced into WT by the floral dip method (Clough and Bent, 1998). Basta-resistant T1 transgenic plants were selected and gene expression was confirmed by genotyping PCR and qRT-PCR (**Supplementary Figures S1D, E**) analyses. T3 generation plants were used for all the experiments. For chromatin immunoprecipitation (ChIP) assay, CDS of *AtbHLH122* and *AtWRKY33* were cloned into pGreen binary vector with hemagglutinin (HA) fusion tag. All the plasmids were sequence verified before use and the primers used in the study are listed in **Supplementary Table S1**.

### RNA Isolation and Quantitative Real-Time PCR (qRT-PCR) Analysis

Total RNA was extracted from leaf and root tissues of control and treated (500 mM NaCl for varying time periods; 0, 1, 3, 6, 12, and 24 h) greenhouse-grown *A. officinalis* and control and treated (50 mM NaCl for varying time periods; 0, 1, 3, 6, 12, and 24 h) tissues of 1-week-old WT *Arabidopsis* seedlings using TRIzol™ reagent (Life Technologies) following the manufacturer’s instructions. An aliquot of this RNA (1 µg) was used to synthesize cDNA using Maxima first strand cDNA synthesis kit for qRT-PCR (Thermo Scientific) following the manufacturer’s instructions. For genotyping and expression analysis of mutants and the heterologous expression lines, DNA and RNA were extracted from leaves of four-week-old seedlings. The qRT-PCR for selected genes was performed as described earlier (Krishnamurthy et al., 2019). The primers used in the study are listed in **Supplementary Table S1**. Constitutively expressed *AtUbiquitin 10* was used as internal control.

### Histochemical GUS Staining

For histochemical study, transcriptional reporter line *pAtKUP2::GUS* was generated. To carry out GUS staining assays, cold stratified T3 seeds were sown on MS plates. One-week-old, untreated and salt treated (50 mM NaCl for 12 h) seedlings and 4-week-old seedling parts were immersed into the GUS staining solution [0.1 M sodium phosphate buffer (pH 7.0), 10 mM EDTA, 0.1% Triton-X, and 2 mM 5-bromo-4-chloro-3-indolyl glucuronide (X-Gluc)]. The tissues were vacuum infiltrated for 2 min and then processed as described (Ravindran et al., 2017). GUS expression in different parts of seedlings was quantified based on the relative intensities of blue coloration using ImageJ software. Data presented are mean ± SE of three biological replicates, each biological replicate consisting of at least six plants.

### Subcellular Localization of AoKUP2 and AtKUP2

The CDS of *AoKUP2* and *AtKUP2* were fused in-frame to both C- and N-terminal of GFP in the *35S::pGREEN* vector and sequence confirmed. Empty vector, *35S::GFP-pGREEN* was used as control. These plasmids as well as the plasmids containing subcellular markers (PM and mitochondria) were introduced into *Agrobacterium*. The subcellular marker plasmids (MT-rk CD3‐991 and PM-rk CD3-1007) were obtained from TAIR. About 3- to 4-week-old leaves of *N. benthamiana* were co-infiltrated with *Agrobacterium* harboring *35S::GFP : At/AoKUP2* or *35S::At/AoKUP2:GFP* and the related subcellular marker constructs. *N. benthamiana* leaf epidermal cells were examined for GFP-Ao/AtKUP2 expression along with mCherry-tagged markers using a confocal laser scanning microscope (Olympus FV3000) at 488 and 561 nm wavelengths, respectively.

### Yeast Strains and Yeast Complementation Assay

*Saccharomyces cerevisiae* strain BY4741 (*MATa his3*Δ*1 leu2*Δ *met15*Δ *ura3*Δ; EUROSCARF) and its derivative: BYT12 (BY4741 *trk1*Δ*::loxP trk2*Δ*::loxP*) (Petrezsélyová et al., 2011) were used to carry out the complementation experiments. Yeast strains were cultivated at 30°C either in standard YPD (1% yeast extract, 2% peptone, and 2% glucose) or the synthetic minimal medium YNB (Difco; 0.67% yeast nitrogen base without amino acids and 2% glucose). Synthetic minimal medium was supplemented with the appropriate auxotrophic requirements. Semi-solid medium was prepared by adding 2% agar. The *Escherichia coli* strain DH5α was used to amplify the plasmid DNA and was grown in Luria–Bertani (LB) broth with 100 μg/mL ampicillin at 37°C. The coding sequence of *AtKUP2* and *AoKUP2* were cloned downstream of the respective promoter into the yeast multicopy vector YEp352 and the primers used for amplification are listed in **Supplementary Table S1**. WT transformed with empty *YEp352* (Hill et al., 1986) plasmid was used as control. The yeast complementation assays with cells expressing *AtKUP2* and *AoKUP2* were performed on semi-solid YNB medium containing appropriate supplements. For drop tests, cell suspensions were adjusted to OD_600_ = 0.2, and 10-fold serial dilutions were made. A 10 μL aliquots of each sample were inoculated onto YNB semi-solid medium containing various concentrations of KCl (500, 1,000, and 1,500 mM) and NaCl (100, 200, and 500 mM). The growth of cells on plates was recorded for 5 days. The growth rate of the transformed WT and *Δtrk1trk2* strains was monitored. These strains were also grown in liquid YPD to log phase (OD_600nm_ = 0.6–0.8). The cells were collected by centrifugation (5,000×g, 10 min) and diluted to an OD_600nm_ of 0.1 prior to culture in liquid SD-Ura with and without salts at 30°C, 160 rpm. The OD_600nm_ was measured every 24 h.

### Seed Germination and Root Length Assay

After surface sterilization, seeds were cold stratified for 3 days at 4°C, before sowing on Murashige-Skoog (MS) agar medium. They were allowed to germinate under 16 h of light/8 h of dark at 22°C. After 1 week of germination, *Arabidopsis* seedlings were carefully removed from MS plate and exposed to salt (50 mM NaCl) treatment. The salt-treated plant tissues were collected at various time intervals (0, 1, 3, 6, 12, and 24 h) and frozen in liquid nitrogen for total RNA isolation. For seed germination assay, the surface sterilized and cold stratified seeds were sown on MS agar plate with and without NaCl treatment (50 and 75 mM) and allowed to germinate as described above. The number of germinated seeds was counted from day one to day four. For root length studies, the sterilized and cold stratified seeds were sown on MS agar plate with and without NaCl, and the root lengths were measured and photographed after 1 week of germination. Similarly, photographs were taken after 14 days for lateral root development assay. For studies in older seedlings, seeds were sown on compost soil and transferred to cold room for 4 days. Trays with cold-stratified seeds were incubated in growth chambers at 23°C and 75% RH under 16 h of light/8 h of dark. Four-week-old seedlings were treated with 150 mM NaCl for 1 week. The soil was rinsed with water twice to remove the soil-bound NaCl followed by a recovery growth in NaCl-free water for 1 week. For cell viability analyses, *Arabidopsis* seeds were germinated on MS plates. Three-day-old seedlings were transferred to MS supplemented with 50 mM NaCl. After the treatment, seedlings were incubated with propidium iodide (pI) for 1 min.

### Estimation of Total Ion Concentration (Na^+^ and K^+^) From Plants

Control and salt-treated 4-week-old *Arabidopsis* plants were harvested and rinsed briefly with distilled water to remove surface contaminating Na^+^. Pools of four plants were taken as one replicate, and at least three independent replicates were used to generate the mean values reported. Leaves and roots from plants were separated at collection and left to dry at 50°C for 2 days. The dried tissue was ground into a powder in liquid nitrogen, and acid digestion and ion analysis were carried out as described earlier (Krishnamurthy et al., 2014).

### Chromatin Immunoprecipitation Using *Arabidopsis* Protoplasts

Mesophyll protoplasts were isolated from leaves of 3- to 4-week-old WT *Arabidopsis* (Col-0) plants and transfected as described earlier (Yoo et al., 2007) with minor modifications. For each transfection, 8–15 μg of purified plasmid DNA (*35S::AtWRKY33* and *35S::AtbHLH122*) was used. Polyethylene glycol (PEG)–CaCl_2_ transfection solution used was as follows: 25% PEG, 0.4 M mannitol, and 150 mM CaCl_2_. The transfected protoplasts were incubated for 20 h at room temperature and fixed with formaldehyde. Protoplasts without any plasmids were used as the negative control. ChIP was performed as described previously (Kaufmann et al., 2010), with minor changes. Anti-HA monoclonal antibody (Santa Cruz Biotechnology) bound to Protein-A agarose beads (Sigma) was used to immunoprecipitate the genomic DNA fragments. ChIP-qPCR analysis was carried out as described (Krishnamurthy et al., 2019).

### Dual Luciferase Assay Using *Arabidopsis* Protoplasts

AtKUP2 promoter fragment of 1.5 kb was cloned into pGreen II-0800-LUC vector. Mesophyll protoplasts isolated from *atwrky33* and *atbhlh22* mutants were transfected with *35S::AtWRKY33* and *35S::AtbHLH122*, respectively. The protoplasts transfected with *pAtKUP2::LUC* construct was used as control. 16–24 h after transfection, protoplasts were pelleted by centrifugation (14,000×*g* for 30 s). Protoplasts were lysed in 1x passive lysis buffer (the Dual-Luciferase^®^ Reporter Assay System, Promega) and incubated at room temperature for 15 min. Following the incubation, 20 μL (approximately 6.6 × 10^4^ cells) of lysed protoplasts were added to 100 μL LARII (the Dual-Luciferase^®^ Reporter Assay System, Promega), vortexed briefly, and measured immediately using GloMax discover (Promega). The luminescence of Luciferase was quenched and Renilla luminescence measured by the addition of 100 μL of Stop & Glo^®^ Buffer (the Dual-Luciferase^®^ Reporter Assay System, Promega) (Iwata et al., 2011). Firefly luciferase activity was normalized to Renilla luciferase activity. Data presented are from five independent biological replicates, each with three technical replicates.

### Statistical Analysis

Data presented are the mean values ± SE/SD. Significance difference between multiple samples was estimated by one-way ANOVA followed by Tukey’s test. Means with same letters are not significantly different, *P* > 0.05. Binary comparisons of data were statistically analyzed by Student’s *t*-test (*P* < 0.05 and *P* < 0.01).

## Results

### AoKUP2 Is Highly Similar to AtKUP*2*

**Figure 1:**
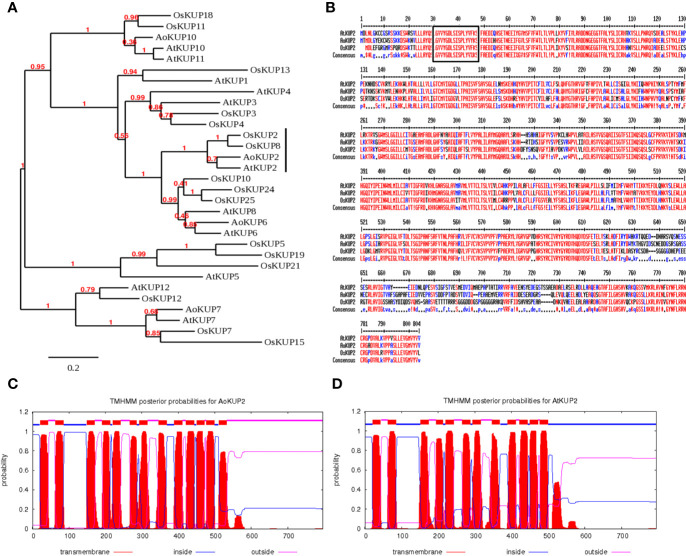
In-silico analysis of AoKUP2 and AtKUP2. **(A)** A phylogenetic tree of KUP family proteins among *A. officinalis*, *A. thaliana*, and *O. sativa*. The phylogenetic tree was constructed using Phylogeny.fr by bootstrap method. The scale bar indicates the branch length. **(B)** Sequence alignment of the deduced amino acid sequences of AoKUP2 with *Arabidopsis* and *O. sativa* KUPs. The black box depicts the characteristic of the first transmembrane fragment, which is conserved in all three transporters. **(C**, **D)** The number of transmembrane domains of AoKUP2 and AtKUP2 were predicted using TMHMM sever 2.0.

Full length coding sequence of *A. officinalis* potassium transporter, *AoKUP2* was obtained from our earlier transcriptomic study (Krishnamurthy et al., 2017). *AoKUP2* cDNA of 2,391 nucleotides in length encodes a polypeptide of 796 amino acids with theoretical pI of 6.74 and molecular weight of 88.32 kDa and its ortholog, the 2,385 bp long *AtKUP2* cDNA encodes a polypeptide of 794 amino acids with theoretical pI of 7.14 and molecular weight of 88.63 kDa. Phylogenetic analysis of KUP family derived amino acid sequences revealed that KUP2 from *Arabidopsis*, *Avicennia*, and rice are grouped into same clade, which suggests that they are homologous to each other (**Figure 1A**). Multiple sequence alignment of deduced amino acid sequences of *AoKUP2* revealed that it has 78% sequence identity and 87% sequence similarity with AtKUP2. Similarly, with *Oryza sativa*, both AtKUP2 and AoKUP2 share about 72% sequence identity and 82% sequence similarity (**Figure 1B**). The number of transmembrane (TM) domains were predicted using TMHMM server, AoKUP2 contains 13 TM helices (**Figure 1C**) and AtKUP2 consists of 12 TM helices (**Figure 1D**). Sequences highlighted with black box (GVVYGDLSISPLY) are characteristic of the first transmembrane fragment, which is conserved in all three classes of K^+^ transporters (AtKUP2, AoKUP2, and OsKUP2).

### Both *Avicennia* and *Arabidopsis KUP2* Are Induced by Salt Stress

**Figure 2:**
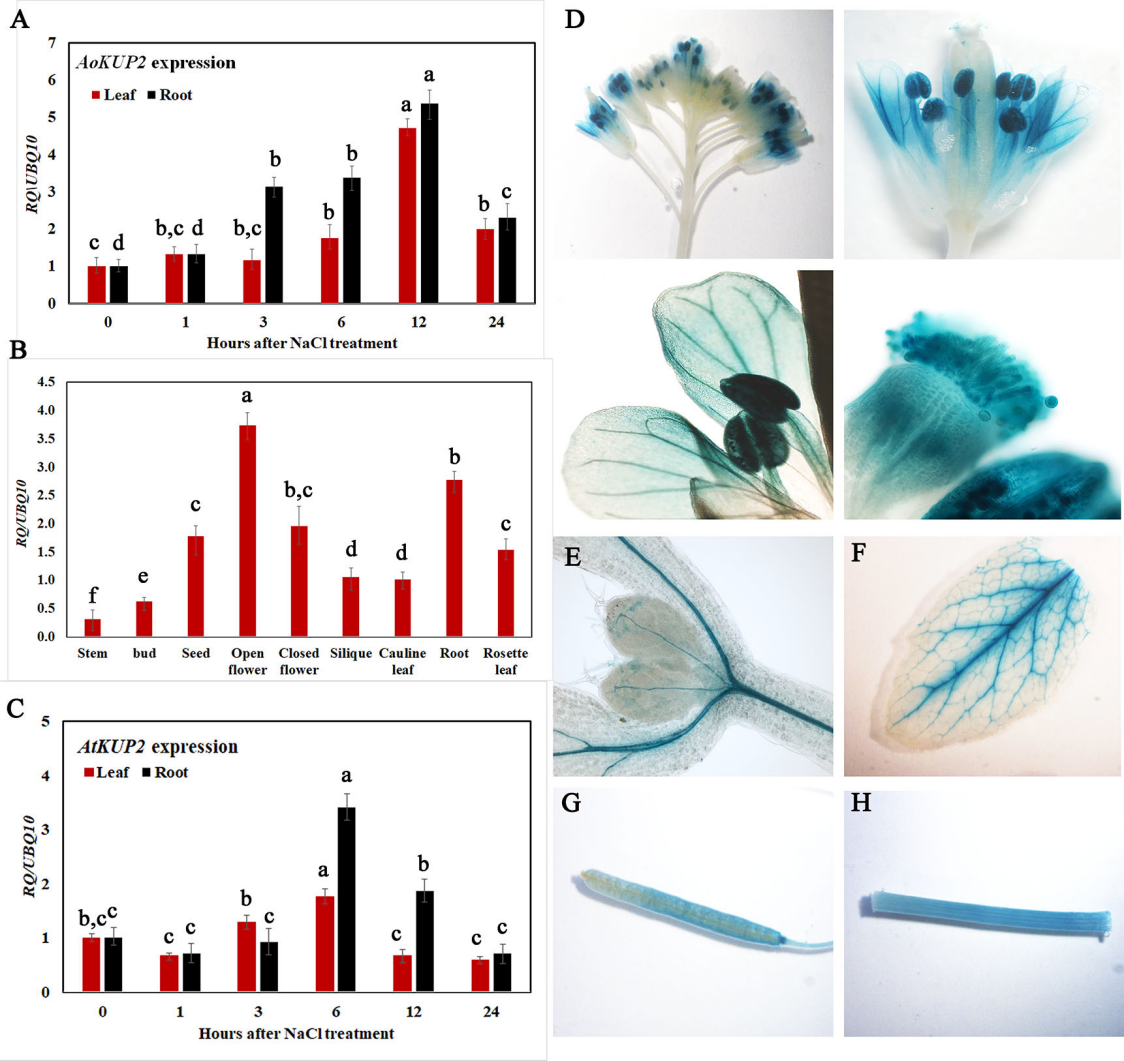
qRT-PCR analysis of *Arabidopsis and A. officinalis KUP2* and GUS expression pattern of *AtKUP2*. **(A)** Time course analysis of *AoKUP2* in 2-month-old *A. officinalis* leaves and root after salt treatment **(B)** tissue-specific expression of *AtKUP2*, **(C)** Time course analysis of *AtKUP2* in 1-week-old *Arabidopsis* leaves and roots after salt treatment. Relative expression levels of transcripts with reference to *Ubiquitin 10* transcript levels are plotted, and qRT-PCR data are mean ± SD from three biological replicates, each with three technical replicates. **(D**–**H)** Tissue specific expression of *pAtKUP2::GUS* in **(D)** open flower, **(E)** cotyledon, **(F)** leaf, **(G)** silique, and **(H)** stem. Different letters in **A** and **C** indicate statistically significant differences between various time points among leaves and roots, as determined by the ANOVA employing the Tukey-Kramer posthoc test (*P* < 0.05). Same letters indicate no statistical difference between them. In **B**, different letters indicate significant differences among various tissues.

To gain insight into the transcriptional responses of *AoKUP2* and *AtKUP2* genes to NaCl stress, expression levels of *AoKUP2 and AtKUP2* were assessed using qRT-PCR after exposure of *Avicennia* and *Arabidopsis* seedlings to salinity for varying durations. The transcript level of *AoKUP2* was induced (~6-fold) after 3 h in roots, and around 5-fold induction was observed in leaves after 6 h of salt treatment (**Figure 2A**). There was a decrease in the expression level of *AoKUP2* after 12 h of salt treatment in both leaves and roots. In *Arabidopsis*, we found that *AtKUP2* is constitutively expressed in all tissue types with the highest level of expression in flowers and roots (**Figure 2B**). Similar to *AoKUP2*, the expression of *AtKUP2* was induced (~4-fold) in roots after 6 h of NaCl treatment, and gradual decrease in expression level was observed after 12 h of treatment (**Figure 2C**). These results suggest that *AoKUP2* and *AtKUP2* might play an important role in salinity.

**Figure 3:**
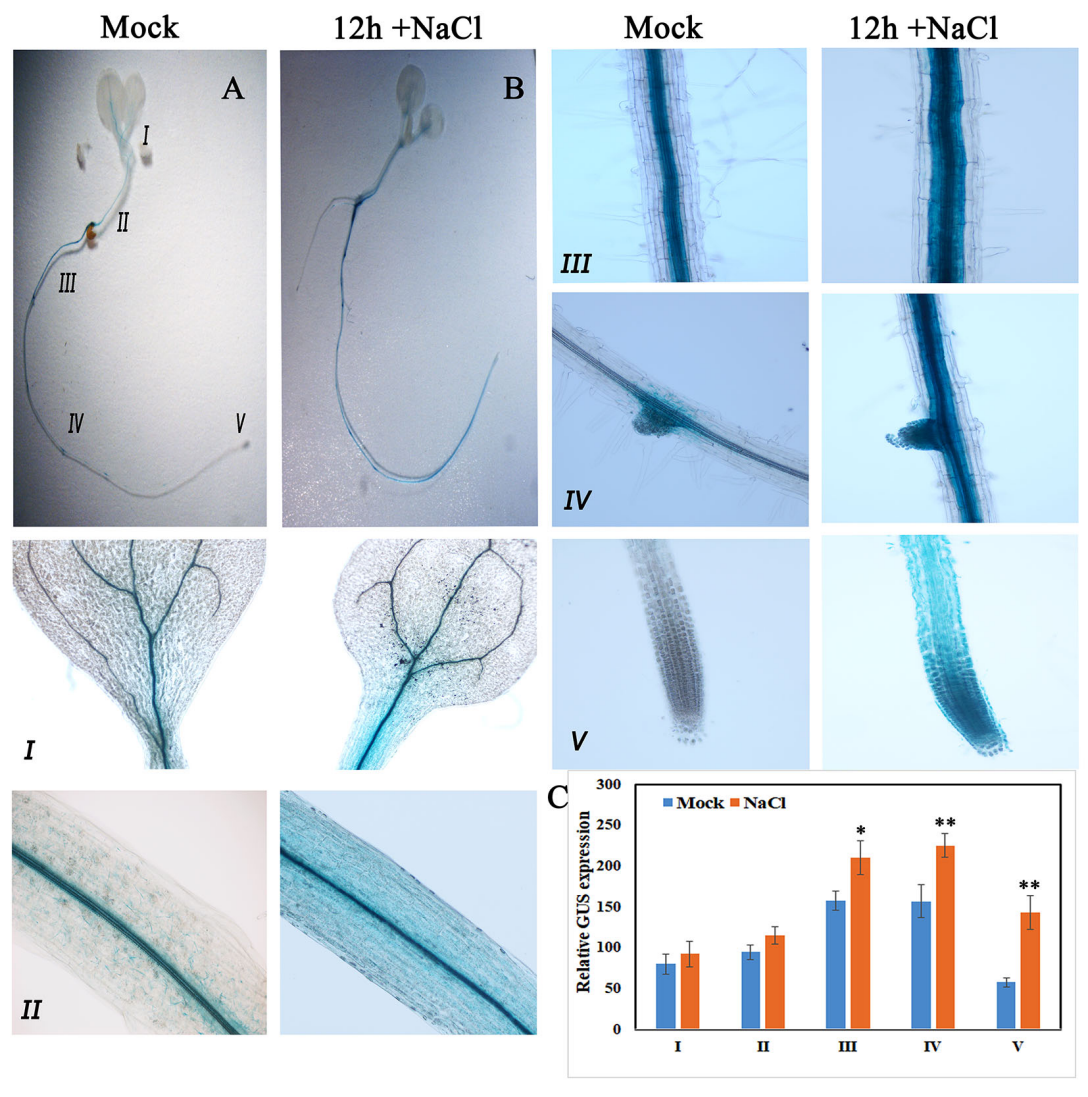
GUS expression pattern of AtKUP2 before and after salt treatment. **(A**, **B)** Tissue-specific expression of *pAtKUP2::GUS* in 1-week-old Arabidopsis seedlings. (I-V) Close-up images of **(A**, **B)** [relative positions labeled in the image **(A)**] before and after 50 mM NaCl treatment for 12 h. **(C)** Relative quantification of GUS intensity in I-V before (mock) and after NaCl treatment. Data are mean ± SE of three biological replicates, each biological replicate consisting of at least six plants. Asterisks indicate statistically significant differences (*P< 0.05 and **P< 0.01) as measured by Student’s *t*-test between mock and the treated.

Consistent with qRT-PCR results, GUS expression driven by the native promoter of *AtKUP2* was also observed in all the plant parts. *AtKUP2* promoter-driven GUS expression was mainly seen in leaf and shoot vasculature, flower, stem, silique, and root stele (**Figures 2D–H**). After salt treatment, no significant difference was observed in the leaves (**Figure 3A**_I), but in the shoots and roots, an increase was seen in GUS expression surrounding the vasculature (**Figure 3A**_II and III). Also, the GUS expression in the root tip and lateral root was significantly increased upon salt treatment (**Figures 3A**_IV and V and **C**).

### Localization of KUP2 in Plasma Membrane and Mitochondria

**Figure 4:**
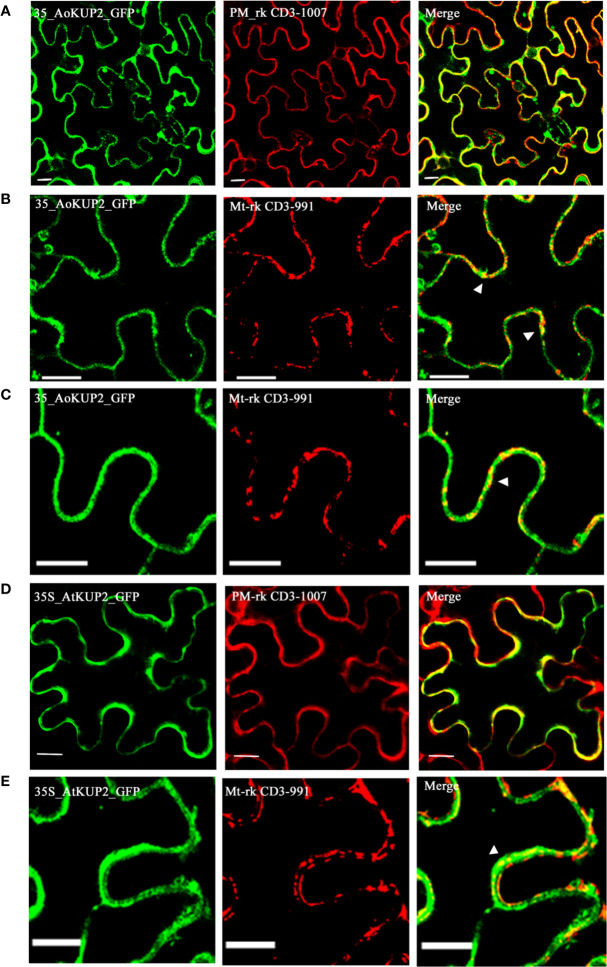
Subcellular localization of AoKUP2 and AtKUP2. Confocal microscopy images of *Nicotiana benthamiana* leaf epidermal cells **(A)** Expression of *35S::AoKUP2:GFP* along with PM marker (PM-rk CD3-1007) and **(B**, **C)** mitochondria marker (mt-rk CD3-991). **(D)** Expression of *35S::AtKUP2:GFP* along with PM marker **(E)** and mitochondria marker. The expression of *35S::AoKUP2:GFP* and *35S::AtKUP2:GFP* were visualized with λ = 488 nm (green) while the expression of PM and mitochondria markers were visualized with λ = 561 nm (Red). Co-localization of *35S::AoKUP2:GFP* and *35S::AtKUP2:GFP* with PM and mitochondria are shown in the merged images. Scale bar = 15 µm.

Although KUP2 transporter is known to be involved in transmembrane transport of K^+^ ions, there is no experimental evidence for its localization in the plant plasma membrane. In order to confirm the localization, *AoKUP*2 and *AtKUP2* cDNAs were cloned into both C-terminal and N-terminal GFP tagged vectors and transiently expressed in *Nicotiana benthamiana* leaf epidermal cells along with PM marker. Both C-terminal and N-terminal tagged fusion proteins showed subcellular distribution pattern of AoKUP2 and AtKUP2 to the plasma membrane (**Figures 4A, D**). In addition, localization of AoKUP2 and AtKUP2 was also observed in the mitochondria (**Figures 4B, C, E**). The arrows (**Figures 4B, C, E**) indicating yellow merged signals show the colocalization of AoKUP2 and AtKUP2 with the mitochondrial marker.

### Expression of *AtKUP2* and *AoKUP2 in S. cerevisiae* Mutant Lacking *TRK* Genes Functionally Complements the K^+^ and Na^+^ Sensitivity

**Figure 5:**
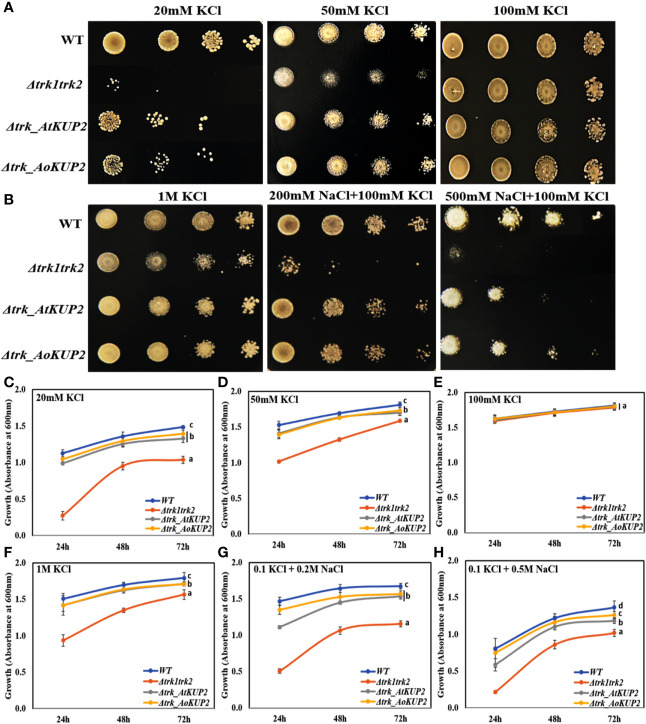
*AtKUP2 and AoKUP2* functionally complement the K^+^ and Na^+^ sensitivity of yeast mutant strains lacking the *TRK1 and TRK2* genes. Growth of BY4741 strain, its derivative BYT12 (*trk1Δ::loxP trk2Δ::loxP*) harboring *AtKUP2* and *AoKUP2* in YEp352 plasmid was tested on SD-Ura medium with **(A)** (10–100 mM KCl) and **(B)** with various salts (1 M KCl, 200 mM NaCl with 100 mM KCl, and 500 mM NaCl with 100 mM KCl). **(C–H)** The density of cells containing *Δtrk1trk2* mutant transformed with *AtKUP2* and *AoKUP2* and WT cells in liquid SD-Ura with various salts (OD_600_) was monitored. Data are mean ± SE of three independent experiments. Means with same letters (shown only for the 72h time point) are not significantly different, *P* > 0.05 (one-way ANOVA followed by Tukey’s test).

In order to carry out functional characterization of AtKUP2 and AoKUP2 transporters, we introduced *AtKUP2* and *AoKUP2* genes into *S. cerevisiae* deletion mutants lacking plasma membrane *TRK* potassium transport system, BYT12 (BY4741 *trk1*Δ*::loxP trk2*Δ*::loxP*). The deletion mutant lacking *TRK* genes and the transgenic yeasts harboring *AtKUP2* and *AoKUP2* did not grow on SD-Ura, while growth of WT (BY4741) harboring *YEp352* vector was not affected. Therefore, they were grown on medium with different concentrations of K^+^ (10 mM to 1 M). All the yeast strains grew well on 100 mM KCl but the mutants could not grow well under low K^+^ (20 and 50 mM) concentrations (**Figures 5A, C–E**). In addition, when KCl concentration was increased to 1 M, growth of the mutant was inhibited but transgenic yeasts harboring *AtKUP2* and *AoKUP2* were not affected (**Figures 5B, F**). Similarly, the mutant displayed sensitivity to NaCl (200 and 500 mM) treatment. But this NaCl sensitivity was rescued in the mutant yeast expressing *AtKUP2* and *AoKUP2* (**Figures 5B, G, H**). These results suggest that KUP2 is involved in K^+^ uptake and might help in maintaining intracellular K^+^/Na^+^ homeostasis which is essential for survival under salt stress.

### Ectopic Expression of *AoKUP2* and *AtKUP2* in *Arabidopsis* Plants Confers Tolerance to Salinity Stress

**Figure 6:**
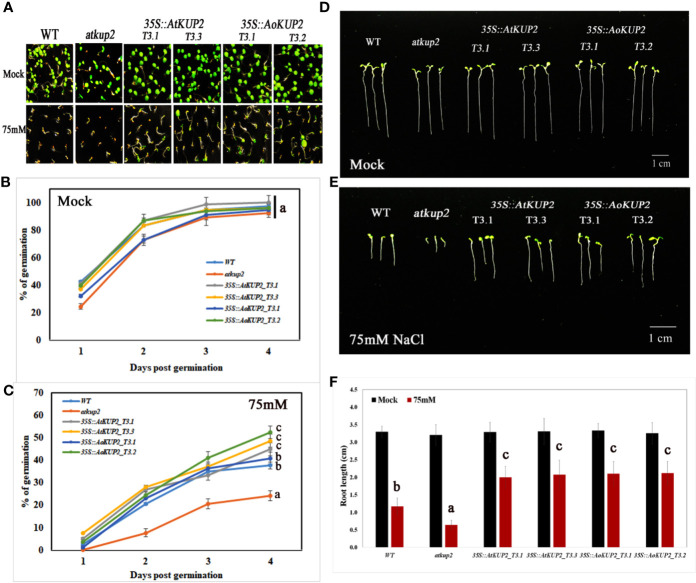
Ectopic expression lines showed reduced sensitivity to NaCl compared to WT and *atkup2*. Germination rate was compared between WT, *atkup2* mutant, and ectopically expressed in WT background (two independent lines each for *35S::AtKUP2* and *35S::AoKUP2*). **(A**–**C)** Germination assay of WT, *atkup2*, *35S::AtKUP2*, and *35S::AoKUP2* lines. The number of germinated seeds were counted from day 1 to 4 and the photographs were taken after 1 week of germination. **(D**–**F)** Comparison of root length among WT, *atkup2*, *35S::AtKUP2*, and *35S::AoKUP2* lines with and without NaCl (75 mM) treatment. Root length measurements and photographs were taken after 1 week of germination. Data are mean ± SE of three independent experiments, each with at least 15 replicates per experiment. Means with same letters are not significantly different, *P* > 0.05 (one-way ANOVA followed by Tukey’s test). Scale bar = 10 mm.

In order to investigate whether ectopic expression of *AoKUP2* and *AtKUP2* in *Arabidopsis* enhances salinity tolerance, WT, mutant, *35S::AoKUP2* and *35S::AtKUP2 Arabidopsis* seedlings were treated with NaCl during both seed germination and vegetative growth stages. Under untreated condition, there was no significant difference in the germination rate of *atkup2* and ectopic expression lines compared to WT. But, cotyledon expansion in *atkup2* was severely inhibited by NaCl treatment, while *35S::AtKUP2* and *35S::AoKUP2* lines showed reduced sensitivity to NaCl stress (**Figures 6A–C**). Around 20–30% decrease in germination rate was observed in the mutant compared to the ectopic expression lines. Similarly, we also examined the root growth in *35S::AtKUP2* and *35S::AoKUP2* lines under salt treatment. Under untreated condition, there was no growth difference between WT, mutant and ectopic expression seedlings. Significant reduction in root length was noted in the mutant and WT seedlings upon 50 mM (**Supplementary Figure S2**) and 75 mM (**Figures 6D, E**) NaCl treatment compared to the ectopic expression lines. *35S::AtKUP2* and *35S::AoKUP2* lines showed about 2.5-fold increase in root length compared to the mutant and ˜1.5-fold increase in root length compared to the WT under 75 mM NaCl treatment (**Figure 6F**).

**Figure 7:**
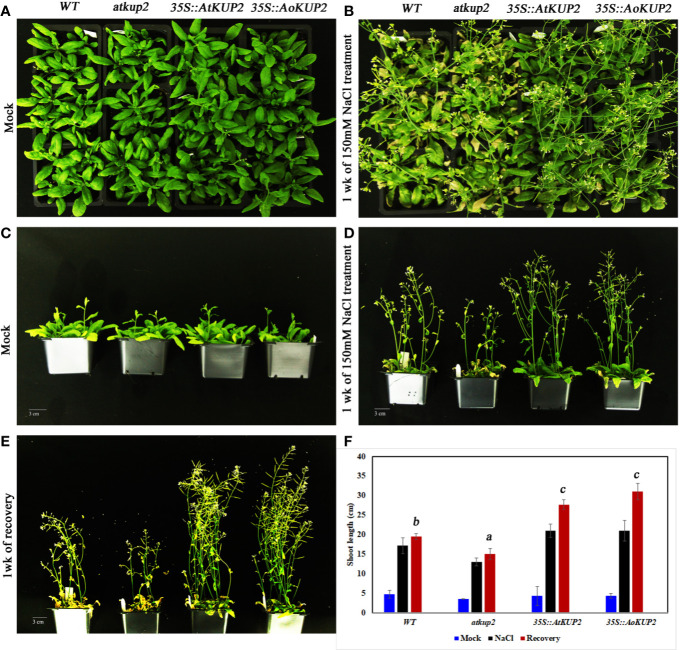
Ectopic expression of *AtKUP2 and AoKUP2* increases salt tolerance of *Arabidopsis* plants. **(A**–**E)** Growth response to salt (150 mM NaCl) was monitored in 1-month-old WT, *atkup2*, and ectopic expression lines (*35S::AtKUP2* and *35S::AoKUP2*) grown on soil. Growth of WT, *atkup2*, *35S::AtKUP2*, and *35S::AoKUP2* lines under **(A**, **C)** untreated and **(B**, **D)** salt treated conditions, **(E)** growth of WT, *atkup2*, and *35S::AtKUP2* and *35S::AoKUP2* lines after recovery growth in normal water for 1 week, and **(F)** shoot length analysis of WT, *atkup2* and *35S::AtKUP2* and *35S::AoKUP2* under treated and untreated conditions. Scale bar = 30 mm. Data are mean ± SE of three biological replicates, each with at least three plants. Means with the same letter within a data set are not significantly different, *P* > 0.05 (one-way ANOVA followed by Tukey’s test). Scale bar = 30 mm.

Salinity tolerance in mature plants grown in the soil was also investigated. One-month-old WT, mutant and ectopic expression *Arabidopsis* plants were treated with 150 mM NaCl for 1 week. WT and mutant plants showed stunted phenotypes with severe chlorosis and eventually died, but the *35S::AtKUP2* and *35S::AoKUP2* plants appeared greener and healthier than WT and mutant (**Figures 7A–C**). Similarly, shoot length assay was also performed under salt treatment and analyzed after recovery. Under salt treatment, WT and mutant displayed reduced growth compared to ectopic expression lines. After 1 week of recovering with normal watering, the WT and mutant could not recover completely, and exhibited short siliques with poor seed set (**Figures 7D–F**). But *35S*::*AtKUP2* and *35S::AoKUP2* lines showed better performance of about 1.5-fold increase in shoot length compared to that of the mutant (**Figure 7F**). These results clearly indicate that *35S::AtKUP2* and *35S::AoKUP2* ectopic expression lines increased the salt tolerance of *Arabidopsis*.

**Figure 8:**
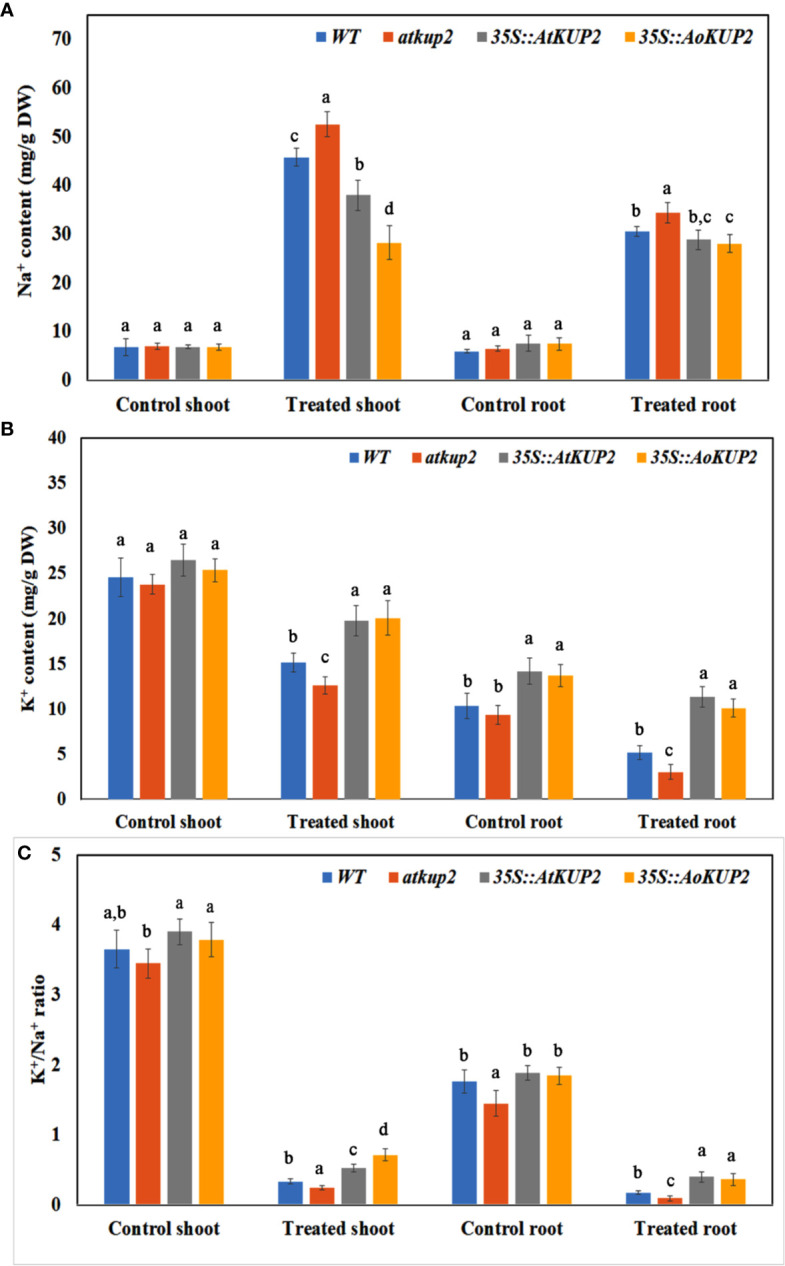
Ectopic expression lines accumulated more K^+^ than WT under NaCl stress. The Na^+^ and K^+^ content in the shoots and leaves of WT, *atkup2*, *35S::AtKUP2*, *and 35S::AoKUP2* lines before and after NaCl treatment. **(A)** Na^+^ content of the shoot and root **(B)** K^+^ content of the shoot and root **(C)** K^+^ to Na^+^ ratio of the shoot and root. Data are mean ± SE of three biological replicates, each with at least three plants. Means with same letter within a data set are not significantly different, *P* > 0.05 (one-way ANOVA followed by Tukey’s test).

In order to examine the K^+^/Na^+^ ratio in plants, we analysed the ion content of WT, mutant, *35S::AoKUP2* and *35S::AtKUP2* plants before and after exposure to NaCl stress. Without NaCl stress, Na^+^ contents were similar in WT, *atkup2* and ectopic expression lines. Upon NaCl treatment, Na^+^ levels increased in all the plants, but the shoot Na^+^ content in the ectopic expression lines was significantly lower (~28 mg/g DW) compared to the WT (~46 mg/g DW) and mutant (~53 mg/g DW) lines (**Figure 8A**). Without NaCl treatment, the K^+^ content did not vary in the shoots of WT, mutant and ectopic expression lines but varied in the roots. Although K^+^ levels decreased upon NaCl treatment in all the plants, the extent of decrease was significantly lower in the shoots and roots of ectopic expression lines compared to that of the WT and mutant (**Figure 8B**). The K^+^/Na^+^ ratio decreased with NaCl treatment in both roots and shoots (**Figure 8C**). However, ectopic expression lines maintained a significantly higher K^+^/Na^+^ ratio than in the WT (~2.5-fold)) and mutant (~3.5-fold) under salt-stressed conditions.

We also investigated the effect of NaCl on lateral root growth and membrane integrity by treating WT, *atkup2* and *35S::AtKUP2* with 50 mM NaCl. Better lateral root growth was observed in *35S::AtKUP2* than WT and *atkup2* under both untreated and treated conditions (**Supplementary Figure S3A**). In untreated condition, no membrane damage was found in WT and *35S::AtKUP2*. But after treatment, the proportion of cell death in the roots gradually increased and comparatively more cell death was observed in *atkup2* and WT than *35S::AtKUP2* (**Supplementary Figure S3B**). This suggests that AtKUP2 might help in preventing general cell damage caused by salinity stress.

### *AtKUP2* Is Regulated by WRKY and bHLH Transcription Factors

**Figure 9:**
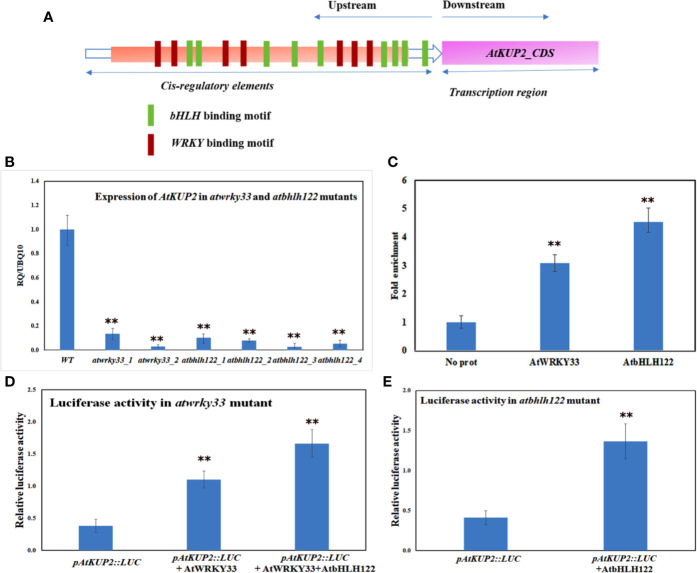
*AtKUP2* is regulated by transcription factors, AtbHLH122 and AtWRKY33 **(A)** Schematic representation of the presence of bHLH and WRKY transcription factors binding motifs in the upstream region of *AtKUP2*. **(B)** Suppression in the transcript levels of *AtKUP2* in the *atbhlh122 and atwrky33* T-DNA insertional mutants compared to WT. **(C)** ChIP-qPCR analysis shows enrichment of *AtKUP2* promoter fragment in AtWRKY33-HA and AtbHLH122-HA ChIP samples. **(D)** Luciferase assay using the mesophyll protoplasts obtained from the leaves of *atwrky33* mutants. **(E)** Luciferase assay using *atbhlh122* mutants. *pAtKUP2::LUC* was used as the reporter and *35S::AtWRKY33* and *35S::AtbHLH122* was used as the effector. Firefly luciferase activity was normalized to Renilla luciferase activity and plotted. Data are mean ± SD of five independent biological replicates, each with three technical replicates. Asterisks indicate statistically significant differences (**P < 0.01) as measured by Student’s *t*-test between WT and the mutants in B and between control and test in **(C, E)**.

Promoter analysis of *AtKUP2* revealed various *cis*-regulatory elements in the 5’ upstream region. The putative binding motifs in the upstream region included *cis*-elements for bHLH and WRKY TFs (**Figure 9A**). By coincidence, in a previous RNA-Seq analysis, bHLH and WRKY were among the major groups of TFs co-induced upon salt treatment in *A. officinalis* roots (Krishnamurthy et al., 2019). Since it is already reported that bHLH and WRKY are regulated by salt stress, we checked the expression of *AtKUP2* in *atbhlh122* and *atwrky33* T-DNA insertional mutants. In *atwrky33*, the expression of *AtKUP2* was suppressed by about 80% and in *atbhlh122*, the suppression level was even higher (~90%) compared to WT (**Figure 9B**). We then carried out ChIP assay to investigate the interaction between *AtKUP2* and bHLH and WRKY transcription factors using *Arabidopsis* protoplasts. AtWRKY33-HA pulldown samples displayed ~3-fold enrichment and AtbHLH122-HA pull down samples displayed ~4-fold enrichment of *AtKUP2* promoter fragment (**Figure 9C**) compared to the vector controls. In addition, we carried out luciferase assay using *atwrky33* and *atbhlh122* protoplasts transfected with *35S::AtWRKY33* and *35S::AtbHLH122*, respectively, together with *pAtKUP2::LUC* in order to check whether AtWRKY33 and AtbHLH122 can activate the expression of *AtKUP2*. *atwrky33* protoplast transfected with AtWRKY33 showed ~3.6-fold higher luciferase activity compared to the control (**Figure 9D**). Similarly, *atbhlh122* protoplasts transfected with *AtbHLH122* showed ~3.4-fold higher luciferase activity compared to the control (**Figure 9E**). We also checked whether both TFs have additive effect on *AtKUP2* expression by co-transfecting both *35S::AtWRKY33* and *35S::AtbHLH122* with *pAtKUP2*::LUC in *atwrky33* mutant (**Figure 9D**). It showed ~5.4-fold higher activity compared to *pAtKUP2*::LUC. These results clearly indicate that AtbHLH122 and AtWRKY33 TFs act together as upstream regulators of *AtKUP2*.

## Discussion

In plants, ionic and osmotic stresses are the two major stresses imposed by soil salinity. Ion transport is a critical step, which controls the ion homeostasis under such stress conditions (Volkov and Beilby, 2017). High affinity KUP/HAK/KT transporters belong to a large K^+^ transporter gene family and have been identified in several crop species including *Arabidopsis, rice* and *maize* (Gupta et al., 2008). Based on our knowledge, KUPs have not been characterized in any of the mangrove species and their heterologous expression has not yet been studied. Mangroves possess efficient salt tolerance mechanisms in order to grow under extreme saline conditions (Kodikara et al., 2018). Therefore, it is possible that their KUPs could function more effectively under salt stress than the corresponding proteins from the non-halophytes. In the present study, we identified a specific *KUP2* from *A. officinalis* that was differentially expressed upon salt treatment (**Figure 2A**). High sequence similarity of AoKUP2 with *Arabidopsis*, *O. sativa* and other plant species show that it may have a similar function in *A. officinalis*. The expression of *AtKUP2* in different tissues of *Arabidopsis* indicated by qRT-PCR (**Figure 2B**) and transcriptional GUS-marker (**Figures 2D–J**) reveals its ubiquitous presence in plants, with higher level of expression in roots and flowers, which is similar to the tissue-specific expression data of HAKs in *O. sativa* (Su et al., 2002; Yang et al., 2014; Chen et al., 2015). The increased expression of *AtKUP2* in the root tissues rather than leaves under salt stress suggests that it may have an important role under such conditions and is similar to the expression pattern of *OsHAK1* under potassium stress conditions (Chen et al., 2015). Increase in *AtKUP2* expression specifically in the younger parts of the roots, and the tissues surrounding root vasculature (**Figures 3A, B**) further suggests its role in regulating influx/efflux of K^+^ ions in order to maintain the intracellular ion homeostasis as reported in earlier studies (Ketehouli et al., 2019; Ragel et al., 2019). There was a significant upregulation of *AoKUP2* in both leaves and roots under salt stress suggesting that *KUP2* might be involved in transport of K^+^ in both leaves and roots under salt stress. Lateral root growth and root elongation are highly affected by limitation of K^+^ (Jung et al., 2009; Kellermeier et al., 2013). The importance of *Arabidopsis* KUP6 in lateral root initiation and development in ABA and auxin signalling pathways is reported (Osakabe et al., 2013). Our observation of enhanced GUS expression in LRP and better lateral root growth in *35S::AtKUP2* in both treated and untreated conditions (**Supplementary Figure S3A**) imply that AtKUP2 has some role in lateral root growth. For better understanding of the underlying mechanism, detailed studies need to be carried out. In addition, localization of AoKUP2 and AtKUP2 in plasma membrane (**Figure 4**) could be required for increased transport of K^+^ across the root plasma membrane in order to maintain intracellular K^+^/Na^+^ ratio under salt stressed conditions. The reduced cell damage in the roots of *35S::AtKUP2* compared to WT and *atkup2* under salt treatment (**Supplementary Figure S3B**) further confirms that AtKUP2 might help in preserving the membrane integrity, which is important for survival under salt stress by maintaining a high K^+^/Na^+^ ratio. Also, mitochondrial localization of AoKUP2 and AtKUP2 suggests that they might be involved in ROS cell signalling process during oxidative stress as reported previously for durum wheat mitochondrial K^+^ channel (PmitoKATP) (Pastore et al., 1999; Pastore et al., 2013). Hence, further experiments may help to reveal if these KUPs play any role in the ROS pathway besides salinity tolerance mechanism.

Most of the plant KUP/HAK/KT transporters function in high‐affinity K^+^ transport, although some members from this family were shown to function as low-affinity K^+^ transporters. For instance, *KUP1* from *Arabidopsis* mediates K^+^ uptake at both low and high external supply of K^+^ in *Arabidopsis*‐suspension cells and yeast (Ahn et al., 2004; Ketehouli et al., 2019). In the current study, we show that the expression of *AtKUP2* and *AoKUP2* in the mutant yeast strain, *trk1*Δ*trk2*Δ could rescue the growth defect at both low (50 mM) and high external (1 M) K^+^ conditions. Similarly, expression of *OsHAK2*, *OsHAK7* and *HvHAK2* rescued the sensitive phenotype of mutant *E. coli* strain with the defect of K^+^ uptake under low K^+^ supply (Mangano et al., 2008; Alemán et al., 2009; Nieves-Cordones et al., 2010; Horie et al., 2011). Our results show that the growth suppression of the yeast mutants in the 500 mM NaCl with 100 mM KCl medium could be rescued by the introduction of *AtKUP2* and *AoKUP2* (**Figure 5**) suggests that KUP2 mediates K^+^ transport (and could regulate K^+^ and Na^+^ homeostasis) under NaCl stress in yeast cells. We can speculate that it may function in a similar manner in the plants. Several important Na^+^ transporters such as NHXs, SOS1 and HKTs found in glycophytes as well as in some halophytes are known to mediate salt resistance in plants (Horie et al., 2009; Yang et al., 2009; Mishra et al., 2014; Hamamoto et al., 2015; Ma et al., 2019; Al-Harrasi et al., 2020). Ectopic expression of some of these Na^+^ transporters in specific targeted tissues or in whole plants have been reported to increase the salinity tolerance of plants (Yang et al., 2009; Li et al., 2011; Mian et al., 2011; Gao et al., 2012; Huang et al., 2018; Zhang et al., 2019; Al-Harrasi et al., 2020), implying that improvement in detoxification mechanisms of Na^+^ possibly is a typical approach to generate salt tolerant crops. In addition, maintenance of high K^+^/Na^+^ ratio in shoots as wells as roots is essential for salinity tolerance. High-affinity K^+^ uptake was proved to be essential for salinity tolerance in plants (Shabala and Cuin, 2008; Almeida et al., 2017; Wu et al., 2018). The significant increase in salt tolerance of *35S::AtKUP2* and *35S::AoKUP2* lines and increase in shoot and root K^+^/Na^+^ ratio of *35S::AtKUP2* and *35S::AoKUP2* compared to WT and mutant upon salt stress suggests that both AtKUP2 and AoKUP2 mediate K^+^ transport and accumulation during salt stress, which might help in maintaining turgor pressure or membrane potential leading to better survival of plants.

Our computational analysis of *AtKUP2* sequence indicated the presence of various putative TF binding sites recognized as abiotic stress-responsive elements within the promoter region. Amongst these TF binding sites, bHLH and WRKY are abundantly distributed within the promoter region of *AtKUP2* (**Figure 9A**). Our previous study showed that upon salt treatment, the expression of *AtNHX1* and *AtNHX6* are regulated by bHLH TFs, AtbHLH122 and AtMYC2 (Krishnamurthy et al., 2019). Another study reported that overexpressing *Gossypium hirsutum WRKY34* in *Arabidopsis* increased the salt tolerance by developing the plant’s ability for the selective uptake of K^+^ and Na^+^ and maintain high K^+^/Na^+^ ratio in leaves and roots of transgenic plants (Zhou et al., 2015). The suppression of *AtKUP2* expression in *atbhlh122 and atwrky33* mutants coupled with the enrichment of *AtKUP2* promoter fragments in ChIP and enhancement of *AtKUP2* promoter-driven luciferase expression (**Figures 9B, C**) collectively show that AtWRKY33 and AtbHLH122 act as the upstream regulators of *AtKUP2* under salt stress. The regulatory link between *AtKUP2* and the two TFs identified in this study helps to explain a part of the molecular mechanism of action for these different players. Thus, bHLH122 was reported to improve stress tolerance in *Arabidopsis* by reducing the activity of ROS and enhancing the levels of proline (Liu et al., 2014; Liu et al., 2015; Wang et al., 2018; Krishnamurthy et al., 2019). Overexpression of WRKY33 was also shown to improve salt stress tolerance in *Arabidopsis* (Bao et al., 2018). WRKY33 is not only salt-responsive but also is regulated by oxidative stress (Jiang and Deyholos, 2009; Birkenbihl et al., 2012). Furthermore, WRKY33 regulated genes are found to be associated with ROS detoxification mechanisms (Jiang and Deyholos, 2009) suggesting role of WRKY TFs as important regulators in various stress adaptation. Our observations helped to identify the TFs as necessary for the salt-mediated upregulation of *AtKUP2*. So far, there have not been any reports identifying TFs that regulate the expression of *AtKUP2*. Another question that remains to be clarified with further experiments is the nature of interaction between bHLH122 and WRKY33 transcription factors in order to act as a molecular regulator of gene expression.

In conclusion, our data suggest that KUP2 from both *Avicennia* and *Arabidopsis* are induced by salt stress, and ectopic expression of both *AoKUP2* and *AtKUP2* plays an important role in salt remediation of *Arabidopsis*. Our results with heterologous expression of *AtKUP2* in selected yeast strains show that it complements the function of plasma membrane K^+^ transport and in Arabidopsis helps to maintain higher K^+^/Na^+^ ratio. In addition, using ChIP and luciferase, we show that transcriptional regulation of *AtKUP2* occurs by AtWRKY33 and AtbHLH122. In future, studies on KUP2 need to be carried out in crop plants such as rice and wheat in order to produce salt tolerant crop plants that will help to address food security despite the increasing soil salinization occurring globally.

## Data Availability Statement

All datasets presented in this study are included in the article/**Supplementary Material**.

## Author Contributions

SR, PK, and PPK conceived the research plans. SR and PK designed the experiments. SR carried out all the experiments, analyzed the data, and wrote the article with contributions from all the authors.

## Funding

This research grant was supported by a PhD research scholarship to SR from the National University of Singapore.

## Conflict of Interest

The authors declare that the research was conducted in the absence of any commercial or financial relationships that could be construed as a potential conflict of interest.
