# Supplementary material for: Regulation of AtKUP2 Expression by bHLH and WRKY Transcription Factors Helps to Confer Increased Salt Tolerance to Arabidopsis thaliana Plants
Source: Front Plant Sci. 2020 Aug 25;11:1311. doi: 10.3389/fpls.2020.01311 (PMC7477289; doi:10.3389/fpls.2020.01311)
Supplement: Supplementary file 1 [file DataSheet_1.pdf]

Online supplementary material for

Regulation of *AtKUP2 expression* by bHLH and WRKY transcription factors helps to confer increased salt tolerance to *Arabidopsis thaliana* plants

Sivamathini Rajappa[1], Pannaga Krishnamurthy[2] and Prakash P Kumar[1,2,*]

[1] Department of Biological Sciences, National University of Singapore, 14 Science Drive 4, Singapore 117543

[2] NUS Environmental Research Institute (NERI), National University of Singapore, #02-01, T-Lab Building, 5A Engineering Drive 1, Singapore 117411

***** Corresponding author

Address: Department of Biological Sciences, National University of Singapore, 14 Science Drive 4, Singapore 117543
Tel: +65-65162859
Fax: +65-67792486
email: dbskumar@nus.edu.sg

Supplemental Figure S1

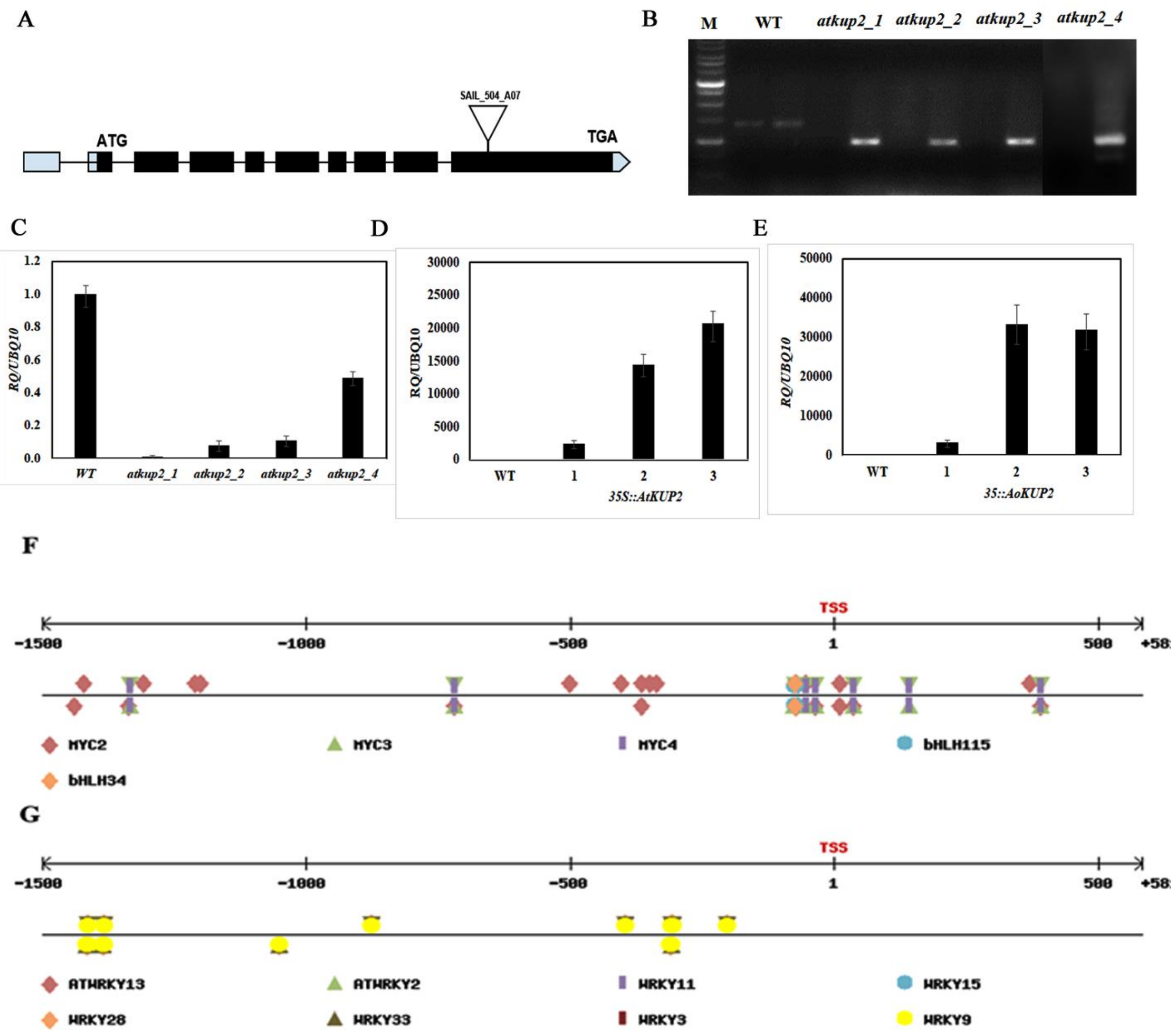

Supplemental Figure S1

Details of *Arabidopsis* mutant and qRT-PCR analysis of mutants and ectopic expression lines: (**A**) Genetic map of *AtKUP2* T-DNA insertion (**B**) Genotyping of *atkup2* shows the homozygous T-DNA insertion **(C)** qRT-PCR shows suppressed expression of *AtKUP2* in *atkup2* T-DNA insertional mutants (**D and E**) qRT-PCR shows high expression of *35S::AtKUP2* and *35S::AoKUP2* in WT background, respectively. Relative expression levels of transcripts with reference to *Ubiquitin 10* transcript levels are plotted, qRT-PCR data represent means ± SD **(F and G)** PLANTPAN2.0 promotor analysis of *AtKUP2* shows the presence of bHLH and WRKY binding motifs.

Supplemental Figure S2

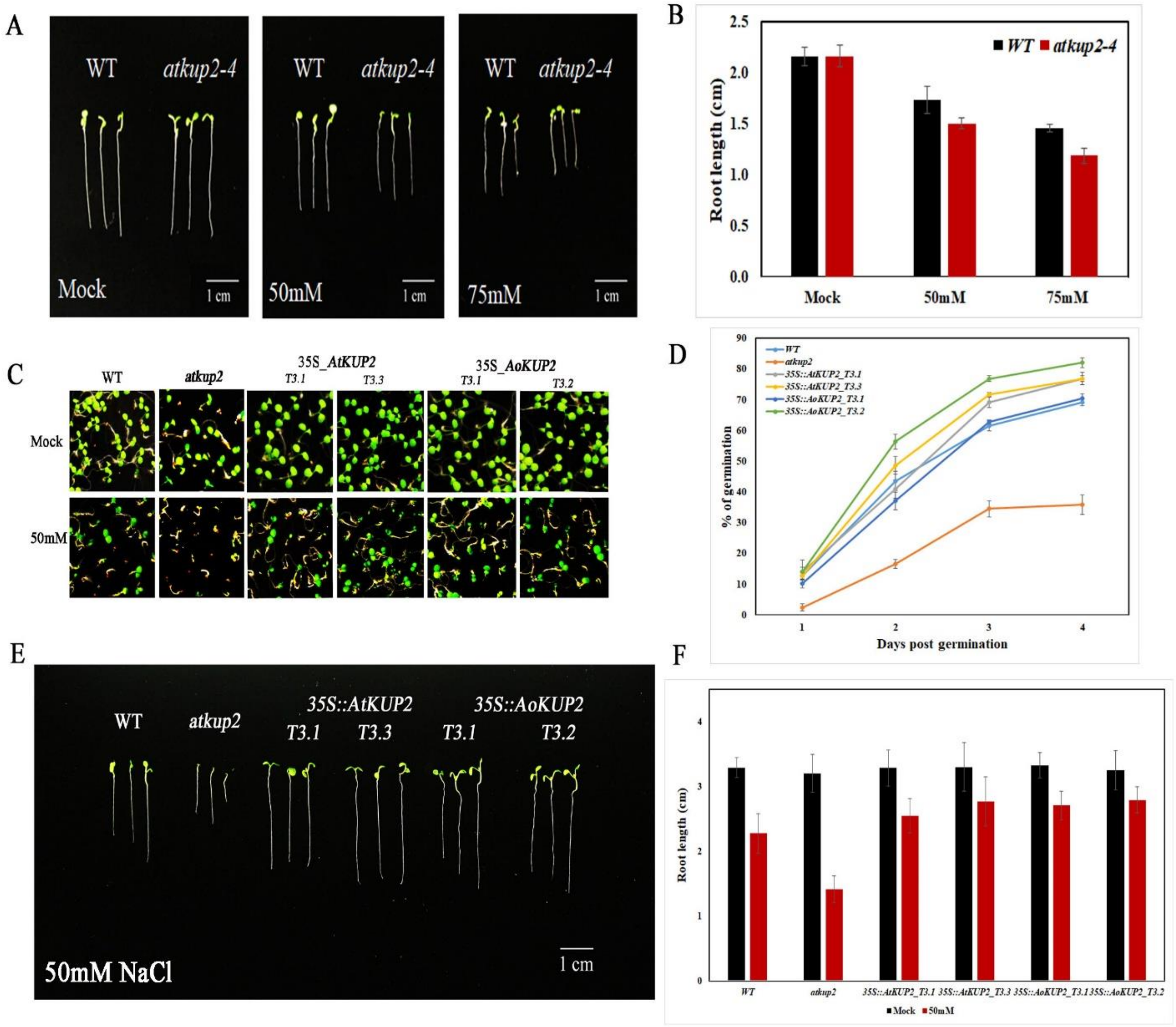

Supplemental Figure S2

Responsiveness of WT, *atkup2* and transgenic *Arabidopsis* lines to NaCl treatment. (A and B) Root length assay of *atkup2-4* **(C and D)** Germination assay of WT, *atkup2*, *35S::AtKUP2* and *35S::AoKUP2* grown under 50 mM NaCl condition (**E and F**) Root length assay of WT, *atkup2*, *35S::AtKUP2* and *35S::AoKUP2* lines grown under untreated and treated (50 mM NaCl) conditions. Scale bar=10 mm.

Supplemental Figure S3

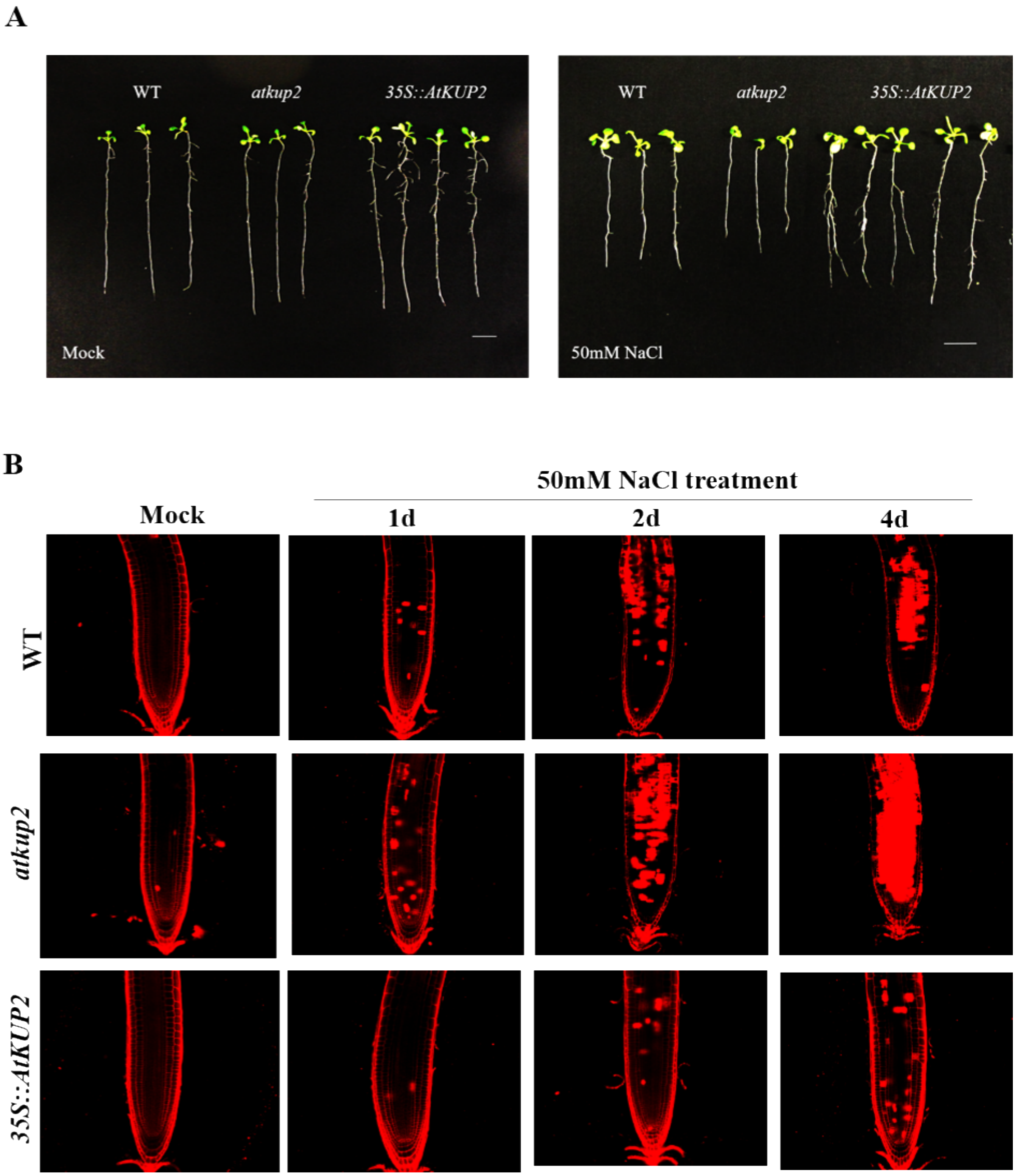

Supplemental Figure S3

Lateral root growth and cell viability test (A) Lateral root assay of WT, *atkup2* and *35S::AtKUP2* grown under mock and 50 mM NaCl condition, Scale bar = 10 mm **(B)** Three-day-old seedlings were transferred to MS agar medium containing 50 mM NaCl and grown for 1- 4 days, followed by propidium iodide (PI) staining.

Supplemental Table S1

Primer sequences used in the study

**Primers**	**Forward**	**Reverse**
For cloning *AoKUP2* in 35S::pGreen vector	AAG CTT ATG ACA ATG GAT CTT GGC TAT GAG AAG	CCC GGG CAC CAC ATA AAC CAT
For cloning *AtKUP2* in 35S::pGreen vector	AAG CTT ATG GAT CTC AAT CTC GGA AAA TGC TG	CCC GGG AAC AAC ATA AAC CAT GCC
For cloning *AtKUP2* in YEP352 vector	CCC GGG ATG GAT CTC AAT CTC GG	AAG CTT TCA AAC AAC ATA AAC CAT GCC GAC T
AtKUP2 qRT primers	CATAACTGGTGCTGAGGCCA	AAGCAGCTTGCCCCATGTAT
AoKUP2 qRT primers	GGACTGCTGACTCCTGCAAT	AGCCAACACTATCGGTGCA A
For cloning *proAtKUP2* in pGreen::GUS vector	GAA TTC TTC CAA GCA AAA TCT TGA TGA TAT AAG AGA C	GGA TCC CGT TTT CTC GGG G
ChIP Primer: qPCR_*KUP2*_WRKY1	AGTGAGCTTCACATATCAACAG TG	GCTGTCTTTTCAGTTTGGTGT
ChIP Primer qPCR_*KUP2*_bHLH1	ACACACTAAAACAATTCAGAG	ATTTAACACATGTCTTTATTT GTA

ChIP Primer qPCR_*KUP2*_WRKY2	AAATATATTATTGTTGTTGACTGA	TTTTCCGAAACTTTTTGTTGC
ChIP Primer qPCR_*KUP2*_bHLH2	TGTAACTGTCGGGAGAGCAC	GAAAGAGAGCAAACGTGGCG
Genotyping primers	TCAGGCAAAGAACAAAGAAGC	CCAGTGGCTCAACTTGAAGAC
For cloning *AoKUP2* in 35S::pGreen:GFP vector	AAG CTT ATG ACA ATG GAT CTT GGC TAT GAG AAG	CCC GGG CAC CAC ATA AAC CAT
For cloning *AtKUP2* in 35S::pGreen:GFP vector	AAG CTT ATG GAT CTC AAT CTC GGA AAA TGC TG	CCC GGG AAC AAC ATA AAC CAT GCC
